# An update on the clinical trial research of immunotherapy for glioblastoma

**DOI:** 10.3389/fimmu.2025.1582296

**Published:** 2025-05-02

**Authors:** Yichen Zhou, Fanxing Shi, Junyu Zhu, Yi Yuan

**Affiliations:** ^1^ School of Elderly Care Services and Management, Nanjing University of Chinese Medicine, Nanjing, Jiangsu, China; ^2^ School of Medicine, Nanjing University of Chinese Medicine, Nanjing, Jiangsu, China; ^3^ Nanjing University of Chinese Medicine, Nanjing, Jiangsu, China

**Keywords:** glioblastoma, immune checkpoint inhibitors, CAR-T, tumor vaccine, oncolytic virus therapy, PD-1 inhibitors, CTLA-4 inhibitors, DC vaccines

## Abstract

Glioblastoma multiforme (GBM) is a common primary malignant brain tumor in adults, characterized by a high rate of recurrence and mortality. The median overall survival is less than 2 years with the current standard therapy. As immunotherapy has begun to show promising results in solid tumors such as non-small cell lung cancer and melanoma in recent years, immnunotherapy for patients with glioblastoma is also in full swing, which is mainly consisted of immune checkpoint inhibitors, cancer vaccines, chimeric antigen receptor T-cell and oncolytic viral therapy. However, the application of immunotherapy in glioblastoma is severely hampered by cognitive impairment of intracerebral lymphatic system, the existence of blood-brain barrier, highly immunosuppressive tumor microenvironment and GBM’s intrinsic features, including low tumor mutation burden and high heterogeneity. This review systematically evaluates recently published clinical trial outcomes of GBM immunotherapy, critically analyses both the progress and limitations of these trials, thoroughly examines current barriers to effective immunotherapy, and highlights promising preclinical studies that may guide future therapeutic development.

## 1 Introduction

Glioblastoma represents the most prevalent primary malignant brain tumor in adults (1), comprising 50.1% of all primary malignant central nervous system (CNS) neoplasms (2). With an overall incidence rate of approximately 3.22 cases per 100,000 individuals, its occurrence demonstrates both age-dependent progression and male predominance (3). GBM has consistently been characterized by its rapid disease progression, elevated mortality rates, unfavorable clinical outcomes, and frequent tumor recurrence (4). The 2021 World Health Organization (WHO) classification of CNS tumors categorizes glioblastoma as a grade IV, IDH wild-type adult diffuse glioma, reinforcing its recognition as an exceptionally aggressive malignancy with dismal prognosis (5).

Currently, the standard treatment for newly diagnosed glioblastoma (ndGBM) involves maximal safe surgical resection followed by concurrent chemoradiotherapy (CCRT), with subsequent adjuvant temozolomide (TMZ) and tumor treating fields (TTF). However, no consensus treatment exists for recurrent GBM (rGBM) (3). Alternative options include DNA damage response (DDR) inhibitors and targeted molecular therapies. Despite these treatments, the median overall survival (mOS) remains limited to approximately 20.9 months, with nearly universal tumor recurrence (6).

The high recurrence rate stems from multiple factors: the infiltrative growth pattern of GBM prevents complete surgical removal (7); the blood-brain barrier restricts delivery of large molecules and poorly lipid-soluble drugs (8) and the prevalent emergence of resistance to radiotherapy develops through various mechanisms, including the presence of quiescent glioma stem cells (GSCs), protective peritumoral brain (PTB) tissue (9) and intact DNA repair systems in O6-methylguanine-DNA methyltransferase(MGMT) unmethylated gliomas (10). The low tumor mutation burden and high degree of tumor heterogeneity present significant challenges for targeted molecular therapies. Furthermore, the profoundly immunosuppressive tumor microenvironment (TME) contributes to diminished T-cell infiltration and function through multiple mechanisms, including upregulation of inhibitory immune checkpoints (11), HIF-1α-mediated lactate accumulation (12, 13) and elevated TGF-β expression (14), collectively rendering GBM an immunologically "cold" tumor. These limitations underscore the critical need for novel therapeutic approaches, with immunotherapy emerging as one of the most promising strategies for GBM patients.

The concept of immunotherapy dates back to 1890 when Dr. William Coley pioneered the use of bacterial injections for treating inoperable malignancies. However, its application in glioblastoma faced considerable delays owing to the prevailing belief in the brain's "immune-privileged" status (15, 16). This scientific paradigm remained unchallenged until the 20th century when the discovery of the brain's dural lymphatic system fundamentally altered our understanding of CNS immunity (17). Moreover, clinical observations of immune-mediated neurological pathology in patients with autoimmune inflammatory diseases (18) and neurodevelopmental disorders (19, 20) provided definitive evidence for the bidirectional communication between central nervous system immunity and peripheral immune function. These seminal findings revolutionized neuroimmunological concepts and established the scientific foundation for developing immunotherapies against glioblastoma.

Immunotherapy for glioblastoma has evolved to encompass both established and emerging modalities. The classical approaches, including immune checkpoint inhibitors and cancer vaccines, function indirectly by stimulating the host immune system to identify and destroy tumor cells (21). Recent biotechnological advances have introduced more direct therapeutic strategies such as T cell therapies and oncolytic viruses. Currently, multiple immunotherapies for GBM have advanced to Phase II/III clinical trials, which can be systematically classified into four categories according to their mechanisms: immune checkpoint inhibitors, cancer vaccines, chimeric antigen receptor T-cell (CAR-T), and oncolytic viral therapy.

This review offers a thorough evaluation of recent clinical progress across these four principal immunotherapeutic strategies for glioblastoma. We perform a critical assessment of existing limitations in immunotherapy development and provide insightful perspectives on future therapeutic directions for GBM management.

## 2 Immune checkpoint inhibitors

For decades, it has been well-established that tumor cells evade anti-tumor immune responses by overexpressing inhibitory immune checkpoints (e.g., PD-1, PD-L1). These checkpoints interact with their respective ligands on T lymphocytes, effectively suppressing T-cell activation and enabling immune escape (22). To counteract this immunosuppressive mechanism, several immune checkpoint inhibitors targeting PD-1, PD-L1, CTLA-4 and other ones have been developed and implemented in clinical practice (**Table 1**), such as Nivolumab, Pembrolizumab, and Cemiplimab (23–26).

**Table 1:** Summary of Immune checkpoint inhibitors clinical trials in glioblastoma.

Author	Study Design	Invention	Control	NCT NO	Number of patients	Conditions	Lessons learned (intervention vs. control)
Reardon et al. (28)	III RCT	NIVO	BEV	NCT02017717	369	rGBM	mOS 9.8 vs. 10.0 months; mPFS 1.5 vs. 3.5 months
Lim† et al. (29)	III RCT	NIVO,SOC	SOC	NCT02667587	716	ndGBM MGMT methylated	mOS 28.9 VS 32.1 months; mPFS 10.6 VS 10.3 months
Omuro et al. (30)	III RCT	NIVO,RT	SOC	NCT02617589	560	ndGBM MGMT unmethylated	mOS 13.4 vs. 14.9 months; mPFS 6.0 vs. 6.2 months; 24-month OS rates 10.3% vs. 21.2%
Nayak et al. (33)	II RCT	BEV,PEM	PEM	NCT02337491	80	rGBM	mOS 8.8 vs. 10.3 months; mPFS 4.1 vs. 1.43 months
Iwamoto et al. (36)	II RCT	BEV,PEM,RT	PEM,RT	NCT03661723	60	rGBM	mOS 7.6 vs. 11.5 months; mPFS 4.14 months vs. 4.9months
Groot et al. (41)	II Single arm	Neoadjuvant PEM	NA	NCT02337686	15	rGBM	mOS 20.3months; mPFS 4.5months
Campian et al. (42)	II RCT	LITT, PEM	PEM	NCT02311582	34	rGBM	mOS 11.4 vs. 5.2months; mPFS 10.5 vs. 2.1 months
	II RCT	HSPPC-96,PEM,SOC	PLACEBO,PEM,SOC; PEM,SOC	NCT03018288	32	ndGBM	mOS 14.4 vs. 14.1 vs. 10.1months; mPFS 13.7 vs. 8 vs. 10.3months
Tran et al. (44)	II Single Arm	PEM,TTF,SOC	Historical Control	NCT03405792	26	ndGBM	mOS 24.8 versus 14.7 months
Sloan et al. (47)	I Single Arm	IPI,NIVO,SOC	NA	NCT02311920	32	ndGBM	mOS 20.7 months; mPFS 16.1 months
Lassman et al. (48)	II RCT	NIVO, IPI, RT	SOC	NCT04396860	159	ndGBM MGMT unmethylated	mOS around 13 months vs. around 13 months; mPFS 7.7 vs. 8.5 months
Omuro et al. (49)	I RCT	NIVO,IPI	NIVO	NCT02017717	30	rGBM	mOS: 7.3 vs. 10.4 months; mPFS: 2.1 vs. 1.9 months

RCT, randomized controlled trial; TMZ, temozolomide; NIVO, Nivolumab; BEV, bevacizumab; rGBM, recurrent glioblastoma; ndGBM, newly diagnosed glioblastoma; mOS, median overall survival; mPFS, median progression-free survival; SOC, Standard of Care, including surgical resection, concurrent chemoradiotherapy, adjuvant temozolomide for ndGBM; PEM, pembrolizumab; RT, radiotherapy; LITT, laser interstitial thermal therapy; TTF, tumor treating fields; IPI, ipilimumab. All cited literature consists of peer-reviewed publications or registered clinical trials from the ClinicalTrials.gov database.

### 2.1 Nivolumab

Nivolumab, a common PD-1 immune checkpoint inhibitor, exerts its antitumor effect by binding to PD-L1 on tumor cells, thereby blocking the PD-1-mediated tyrosine phosphorylation signal that inhibits T-cell activation (27).

CheckMate 143 demonstrated that nivolumab monotherapy failed to show a statistically significant overall survival benefit compared to bevacizumab (Bev) in the overall rGBM population (28). Nevertheless, subgroup analysis revealed a potential survival advantage for nivolumab in patients with MGMT methylation.

For ndGBM patients, neither nivolizumab combined with standard of care (SOC) in MGMT-methylated cases (29), nor nivolizumab combined with radiotherapy alone in MGMT-unmethylated cases (30) demonstrated significant improvement in median overall survival compared to SOC, which was defined as maximal safe resection followed by CCRT and adjuvant TMZ according to 2016 WHO Classification of Tumors of the Central Nervous System (31).

This limited efficacy may be attributed to chemotherapy or RT induced T-cell depletion (32) and suppression of T cell function as a consequence of suppressive immune microenvironment and low immunogenicity of GBM. Supporting evidence comes from a phase II clinical study conducted by Schalper et al (32), where immunohistochemical analysis revealed that while nivolumab treatment increased intratumoral immune cell infiltration likely mediated by elevated chemokine levels, it failed to significantly enhance either T-cell cytotoxic activity or proliferative capacity.

To maximize the clinical benefit of nivolumab, a multifaceted optimization strategy is required. First, comprehensive pharmacodynamic studies are needed to validate the effects of intravenous nivolumab on tumor-infiltrating T-cell activity *in vivo*. Second, treatment combinations should be carefully considered with chemoradiotherapy. Third, precision patient selection through refined enrollment criteria is crucial, as evidenced by differential treatment responses between nivolumab and bevacizumab in patient subgroups stratified by PD-L1 expression levels (28). Notably, nivolumab demonstrates particular efficacy in the B7-H4-high glioma subgroup - a population intrinsically characterized by profoundly depleted tumor-infiltrating lymphocytes (TILs) (33).

### 2.2 Pembrolizumab

Originally developed for unresectable or metastatic melanoma (34), the PD-1 antagonist pembrolizumab has shown modest activity in recurrent glioblastoma, with phase II randomized controlled trials reporting a median overall survival of 10.3 months for monotherapy (35) and 11.5 months when combined with radiotherapy (36). However, combination strategies with bevacizumab—including both Pem+Bev and Pem+RT+Bev regimens—paradoxically compromised survival outcomes (35, 36). This observed reduction in therapeutic efficacy may stem from increased treatment-related adverse events due to Bev-associated toxicity (35, 37, 38) and Bev-mediated exacerbation of tumor hypoxia, which may further potentiate the immunosuppressive tumor microenvironment (39, 40).

Surprisingly, neoadjuvant pembrolizumab has demonstrated efficacy in recurrent glioblastoma, with a phase II trial showing prolonged survival through preoperative tumor reduction (41). This promising outcome has prompted an ongoing phase IV confirmatory study (NCT05235737, completion May 2026).

Further enhancing this approach, a separate phase II randomized controlled trial (RCT) revealed that laser interstitial thermal therapy (LITT) synergizes with Pem, significantly improving median survival (42) via blood-brain barrier disruption and pro-apoptotic effects (43). Building on these findings, an active phase II trial is currently evaluating a multimodal regimen combining tumor-treating fields, Pem and LITT (NCT06558214).

For newly diagnosed glioblastoma, phase II RCT data indicate a median survival of 10.1 months with pembrolizumab plus SOC (NCT03018288), though this may attribute to the limited 26-month follow-up. More promisingly, Pem combined with maintenance TMZ and tumor-treating fields after SOC demonstrated significantly improved median survival (25.2 months) versus historical controls (44). These results have propelled an ongoing phase III trial evaluating this triple combination (NCT06556563), with completion anticipated by April 2029.

### 2.3 Ipilimumab

CTLA-4 is a negative co-stimulatory molecule expressed on activated T cells. Due to its high homology with the extracellular domain of the T cell co-stimulatory molecule CD28, and its higher affinity for CD80/CD86, CTLA-4 often inhibits T cell activation by competitively binding to CD80/CD86 (45). Additionally, it can reduce the ex-pression of CD80/CD86 on the surface of antigen-presenting cells through trans-endocytosis (46). Ipilimumab, the most clinically established CTLA-4 inhibitor, counteracts these immunosuppressive effects.

For newly diagnosed GBM, a phase I trial demonstrated promising mOS of 20.7 months with ipilimumab, nivolumab on the basis of SOC, though requiring phase II validation (47). In MGMT-unmethylated ndGBM, the combination of ipilimumab, nivolumab and RT showed comparable mOS but inferior median progression-free survival (mPFS) versus SOC at 13.7-month follow-up (48).

In recurrent GBM, combination of ipilimumab and nivolumab yielded modest mOS of 7.3 months (49), showing no significant advantage over nivolumab monotherapy, warranting cautious evaluation of this combinatorial approach (Nivo, Ipi).

In summary, the limited survival benefit of immune checkpoint inhibitor monotherapy primarily stems from the profoundly immunosuppressive tumor microenvironment, low tumor mutational burden and inherent immunogenicity of GBM which consequently restrict both quantity and functionality of tumor-infiltrating T cells. Remarkably, the aforementioned TTF and LITT modalities may offer a safer alternative to conventional chemoradiation by promoting tumor apoptosis and tumor-associated antigen (TAA) release, though their precise mechanisms of action require further investigation.

Beyond classical targets, novel immune checkpoints have also entered the phase I / II clinical trial stage,including TIM-3 checkpoint inhibitors (NCT03961971), LAG-3 checkpoint inhibitors relatlimab (NCT02658981 and NCT03493932), TIGIT checkpoint inhibitors ( NCT04656535 ), CD137 checkpoint inhibitors ( NCT02658981) and IDO checkpoint inhibitors ( NCT04047706, NCT02052648).

## 3 Tumor vaccines

Tumor vaccines for glioblastoma can be broadly classified into four main categories: cellular vaccines, protein/synthetic peptide vaccines, nucleic acid vaccines, and viral vector vaccines (**Table 2**). These therapeutic approaches utilize various forms of tumor-specific antigens (TSAs), TAAs or antigen-encoding genes, which are either administered directly or delivered through artificial carriers such as dendritic cells or viral vectors. Following administration, these vaccines aim to fully activate the patient's anti-tumor immune responses, thereby inhibiting tumor growth and progression.

**Table 2:** Summary of tumor vaccine clinical trials in glioblastoma.

Author	Study design	Invention	Control	NCT NO	Number of patients	Conditions	Lessons learned (intervention vs. control)
Liau et al. (57)	III Single Arm	DCVaxL SOC(ndGBM)	ECP	NCT00045968	232; 64	ndGBM; rGBM	mOS 19.3 vs.16.5 months; mOS 13.2 vs. 7.8 months
Weller et al. (66)	III RCT	CDX-110,SOC	KLH,SOC	NCT01480479	745	ndGBM	mOS:17.4 vs. 17.4months; mPFS: 7.1 vs. 5.6 months
Reardon et al. (67)	II RCT	CDX-110, BEV	BEV	NCT01498328	73	rGBM	6-month PFS 28% vs.16%; OS (HR=0.53,95%Cl,0.32-0.88)
Migliorini et al. (69)	I/II Single Arm	IMA950,SOC	NA	NCT01920191	16	ndGBM	mOS 19 months
Bloch et al. (71)	II Single Arm	HSPPC-96,SOC	NA	NCT00905060	46	ndGBM	mOS 23.8 months; mPFS 18 months
Bloch et al. (72)	II Single Arm	HSPPC-96	NA	NCT00293423	41	rGBM	mOS 42.6 weeks; mPFS 19.1 weeks
Bloch et al. (73)	I/II RCT	HSPPC-96,BEV	BEV	NCT01814813	90	rGBM	mOS 7.5 VS 10.7 months
Ahluwalia et al. (76)	II Single Arm	SurVaxM,SOC	NA	NCT02455557	63	ndGBM	mOS 25.9 months

RCT, randomized controlled trial; TMZ, temozolomide; BEV, bevacizumab; rGBM, recurrent glioblastoma; ndGBM, newly diagnosed glioblastoma; mOS, median overall survival; mPFS, median progression-free survival; SOC, Standard of Care, including surgical resection, concurrent chemoradiotherapy, adjuvant temozolomide for ndGBM; ECP, external control population; KLH, Keyhole Limpet Hemocyanin. All cited literature consists of peer-reviewed publications or registered clinical trials from the ClinicalTrials.gov database.

### 3.1 DC vaccine

Dendritic cells (DCs), as the body's primary antigen-presenting cells, play a crucial role in antitumor immunity by capturing antigens through phagocytosis, pinocytosis, or receptor-mediated endocytosis, processing them, and presenting antigenic peptides via MHC class I and II pathways to activate CD8+ and CD4+ T cells, respectively (50–52).

In glioblastoma immunotherapy, several DC vaccine platforms have been developed, including DCVax-L (loaded with tumor lysates) (53), GSC-DCV (pulsed with glioma stem cell antigens) (54), ICT-107 (loaded with synthetic peptides targeting glioma stem cell-associated antigens) (55), and CMV-DC (electroporated with CMV pp65 mRNA) (56). While all four approaches begin by isolating and differentiating patient-derived monocytes into immature DCs, they differ in their antigen-loading strategies.

DCVax-L has demonstrated significant clinical benefits in glioblastoma treatment, with phase III trial data showing mOS of 19.3 months for ndGBM patients overall, 30.2 months for MGMT-methylated ndGBM patients, and 13.2 months for rGBM patients (53, 57). The vaccine's personalized manufacturing approach, involving co-culture with patient-derived tumor lysates, enables broad targeting of tumor antigens and effectively addresses GBM's spatial heterogeneity. Although these results showed marked improvement over external controls, the original randomized controlled trial design was complicated by a late-stage crossover, necessitating external control comparisons for analysis. Current investigations include an ongoing phase III randomized trial evaluating DCVax-L combined with radiotherapy specifically in MGMT-methylated glioblastoma patients to further validate these promising findings (NCT03548571).

Additionally, clinical trial data demonstrate that the efficacy of glioma stem cell-targeted DC vaccines is strongly influenced by molecular biomarkers: the phase II single-center RCT of GSC-DCV revealed superior survival benefits in patients with IDH1 wild-type, TERT mutation, and low B7-H4 expression profiles (54), while the phase II multicenter trial of ICT-107 showed significantly improved median progression-free survival in newly diagnosed GBM patients, particularly those with HLA- A1 methylated tumors (55).

### 3.2 Peptide vaccine

Peptide vaccines are the most classic form of cancer vaccines. They consist of TSAs or TAAs that are typically 8 to 30 amino acids in length. These vaccines stimulate an adaptive immune response tar-geting the specific antigen to exert anti-tumor effects (58, 59), including CDX-110,HSPPC-96,SurVaxM and so on.

#### 3.2.1 CDX-110

EGFRvIII, the most common EGFR mutation in glioblastoma with 25-30% prevalence (60), drives tumorigenesis through constitutive activation (61) and promotes radioresistance by enhancing DNA repair capacity (62).

While the EGFRvIII-targeted peptide vaccine rindopepimut (CDX-110) showed promising survival benefits in three phase II trials for ndGBM patients when combined with or without adjuvant TMZ on the basis of CCRT (63–65), these results were not replicated in a subsequent multicenter phase III RCT, potentially due to the unreliability of the results from the previous single-arm clinical trial (66).

Interestingly, in recurrent GBM, rindopepimut combined with bevacizumab demonstrated improved 6-month PFS and OS versus Bev alone (67), suggesting TMZ may compromise vaccine efficacy while Bev's modulation of the immunosuppressive microenvironment (68) could be synergistic.

Anyway, CDX-110, as a single-target peptide vaccine, ultimately demonstrates limited clinical efficacy due to the intrinsic heterogeneity of glioblastoma tumors. This aligns with emerging data on multi-target vaccines like IMA950 (targeting 11 TAAs), which achieved 19-month median survival in ndGBM (69), highlighting the therapeutic advantage of broad antigen targeting over single-epitope approaches.

#### 3.2.2 HSPPC-96

HSPPC-96, a heat shock protein-based vaccine, enhances dendritic cell (DC)-mediated uptake and presentation of tumor antigens by binding to both tumor-derived peptides and CD91 on DCs (70).

In ndGBM, a phase II single-arm trial reported a median survival of 23.8 months with HSPPC-96 following SOC (71). However, a subsequent phase II RCT found no significant survival benefit when combining HSPPC-96 with Pem after SOC (NCT03018288). For rGBM, phase II trials indicate that HSPPC-96—either alone or with bevacizumab—fails to substantially improve long-term survival compared to Bev alone (72, 73). This limited efficacy may stem from GBM’s immunosuppressive microenvironment, which impairs T-cell function, and its inherently low tumor mutational burden.

#### 3.2.3 SurVaxM

Integrin, an anti-apoptotic protein highly expressed on glioma stem cells (GSCs), critically regulates GSCs self-renewal, proliferation, migration, and invasion (9, 74, 75). The survivin-targeting vaccine SurVaxM recently completed a phase II trial in ndGBM patients, demonstrating a median survival of 25.9 months when combined with SOC (76). A phase II RCT (NCT05163080) is now evaluating this regimen.

In summary, GBM's temporal and spatial heterogeneity poses a fundamental challenge for tumor vaccines. Nonetheless, the DCaxL vaccine offers a promising strategy to address spatial heterogeneity. Further exploration of its safety and preparation protocols will be essential if considering its long-term cyclical administration to overcome temporal heterogeneity.

## 4 CAR-T

T cells are central to adaptive anti-tumor immunity, but tumors often evade detection by downregulating MHC expression, suppressing T cell activation (77, 78). Therefore, we engineered chimeric antigen receptors (CARs) on autologous or allogeneic T cells to enable MHC-independent tumor recognition (79, 80). Upon reinfusion into patients, these genetically modified T cells achieve dual therapeutic effects-precise tumor-targeted cytotoxicity and synergistic restoration of tumor-suppressed host immunity (81). For glioblastoma, CAR-T therapies targeting IL13Rα2, EGFRvIII, and HER2 remain in early-phase (I/II) trials (**Table 3**).

**Table 3:** Summary of CAR-T cell clinical trials in high grade glioma.

Author	Study Design	Target	Control	NCT NO	Number of patients	Conditions	Lessons learned (intervention vs. control)
Brown et al. (85)	I Single Arm	IL-13Ra2	NA	NCT00730613	3	rGBM	OS 11 months
Brown et al. (86)	I Single Arm	IL-13Ra2	NA	NCT02208362	65	recurrent grade III/IV glioma	mOS 7.7 months(rGBM); mOS 10.2 months(Tn/mem rGBM)
Brown et al. (88)	I Single Arm	IL-13Ra2	NA	NCT01082926	6	rGBM	no grade 3 or above adverse effects
Rourke et al. (89)	I Single Arm	EGFRvIII	NA	NCT02209376	10	rGBM	mOS 8 months
Goff et al. (90)	I Single Arm	EGFRvIII	NA	NCT01454596	14	rGBM	mOS 6.9 months; mPFS 1.3 months.
Choi et al. (92)	I Single Arm	EGFRvIII WT-EGFR	NA	NCT05660369	3	rGBM	transient tumor regression response in 2 patients; sustained tumor regression response in 1 patient
Bagley et al. (167)	I Single Arm	EGFR IL-13Ra2	NA	NCT05168423	6	rGBM	significant reduction in tumor volume in most patients
Ahmed et al. (96)	I Single Arm	HER2	NA	NCT01109095	17	rGBM	mOS 11.1 months, 1 PR, 7 SD within 29 months

rGBM, recurrent glioblastoma; mOS, median overall survival; mPFS, median progression-free survival; PR, partial response; SD, Stable Disease. All cited literature consists of peer-reviewed publications or registered clinical trials from the ClinicalTrials.gov database.

### 4.1 IL13Rα2

IL13Rα2 expressed in over 50% of GBM patients (82, 83), which has emerged as one of the most promising CAR-T targets for GBM, promotes tumor progression by inhibiting the STAT6 signaling pathway (84). Clinical development has progressed through three generations.

First-generation autologous IL13Rα2 CAR-T achieved 11-month OS in rGBM patients (n=3) via intratumoral delivery (85). However, the clinical significance of these findings remains limited by the small cohort size.

The second-generation IL-13Rα2 CAR-T builds upon the first-generation construct by incorporating the BBζ costimulatory domain and employing a novel dual intratumoral and ventricular delivery approaches combined with the Tn/mem production platform, which enhances undifferentiated memory T-cell populations with favorable marker profiles (increased memory markers, decreased senescence markers. Clinical results demonstrated a median overall survival of 10.2 months in rGBM patients with Tn/mem platform, outperforming Tcm comparator groups (86), effectively verifying the feasibility of the Tn/mem production platform for CAR-T preparation *in vivo* trials.

Although the mOS of second-generation IL-13Rα2 CAR-T cells remained comparable to first-generation therapy from a survival standpoint, imaging assessments revealed more significant benefits.Following 10 weeks of ventricular infusion, one of the patients achieved complete resolution of all detectable intracranial and spinal lesions on both PET and MRI, although disease recurrence occurred at four distinct new locations (87). These observations suggest that the unique tumor-reducing capability of this combined infusion approach in multifocal GBM patients may have important implications for designing future clinical trials.

To address limitations of autologous CAR-T therapy including prolonged manufacturing time and restricted eligibility due to poor T-cell quality, an allogeneic IL-13Rα2 CAR-T product has completed phase I testing (88). The therapy demonstrated a favorable safety profile with no grade ≥3 adverse events or graft-versus-host disease observed, though efficacy evaluation requires further clinical investigation.

### 4.2 EGFRvIII

While EGFRvIII-targeted approaches are well-established in GBM treatment (pioneered by rindopepimut), EGFRvIII CAR-T therapy represents an alternative modality. Phase I trials of intravenously administered EGFRvIII-CAR-T demonstrated limited efficacy in rGBM, with monotherapy achieving median overall survival of 8 months (89). While in another phase I single-arm trial evaluating intravenous infusion of EGFRvIII-CAR-T combined with IL-2, rGBM patients demonstrated not only a limited median survival of just 6.9 months, but also experienced dose-limiting toxicity (90). Most notably, one patient developed pulmonary edema and succumbed to the condition despite resuscitation attempts approximately 4 hours following infusion of 6×10¹° cells.

The observed toxicity profile primarily stems from intravenous CAR-T administration inducing pulmonary vascular toxicity, while the limited clinical efficacy relates to EGFRvIII's special biological behavior whereby the targeted variant co-amplifies wild-type EGFR through paracrine signaling (91) - a mechanistic explanation for both the development of treatment resistance and the frequent detection of wild-type EGFR overexpression in recurrent tumors following EGFRvIII-CAR-T therapy (60, 90).

To address these challenges, Bryan D. Choi's team developed CARv3-TEAM-E T-cells, a dual-targeting approach against both EGFRvIII and wild-type EGFR, delivered via ventricular infusion to circumvent vascular toxicity (92). Initial clinical results demonstrated a manageable safety profile with promising efficacy, including durable tumor regression persisting >150 days after single infusion. However, the preliminary nature of these findings constrained by small sample size and trial design limitations (Single Arm). Thus, larger-scale studies are required to comprehensively evaluate overall and progression-free survival outcomes.

### 4.3 HER2

The HER2 proto-oncogene (17q21) encodes ErbB2, a key EGFR-family tyrosine kinase receptor that is overexpressed in 80% of GBM cases (93). HER2/ErbB2 overexpression drives gliomagenesis through ErbB2 heterodimerization-induced tyrosine autophosphorylation and constitutive activation of proliferative signaling pathways, ultimately promoting uncontrolled cellular proliferation and malignant transformation in glioblastoma (94, 95).

Results from a phase I single-arm trial demonstrated that intravenous HER2-CAR VSTs in rGBM patients achieved a median overall survival of 11.1 months, suggesting potential clinical benefit (96); However, this finding requires validation in larger phase II/III trials.

The therapeutic efficacy of CAR-T cells in GBM remains constrained by late-adaptive resistance mechanisms arising from tumor heterogeneity and the correlation between CAR-T persistence and clinical response continues to be debated. Insights can be drawn from the NY-ESO-1 CAR-T clinical trial for synovial sarcoma and melanoma: the correlation between patients' objective clinical responses and CAR-T persistence was only validated short-term (≤28 days) (97), with stronger association observed for peak peripheral blood CAR-T cell counts (98).

## 5 Oncolytic viral therapy

Oncolytic viruses (OVs) are genetically modified, weakly pathogenic viruses that selectively infect and lyse tumor cells while inducing immunogenic cell death (ICD) in both cancer and stromal cells (99, 100). Their therapeutic potential is mediated through the release of TAAs, damage-associated molecular patterns (DAMPs) from lysed cells, OV-derived pathogen-associated molecular patterns (PAMPs), which collectively mobilize potent antitumor immunity (101–103).

Several engineered viral platforms have been evaluated in clinical trials for glioblastoma, including oncolytic herpes simplex virus, oncolytic adenovirus, poliovirus, retrovirus, Newcastle disease virus (NDV)-based vectors and so on (**Table 4**).

**Table 4:** Summary of oncolytic virus clinical trials in high grade glioma.

Author	Study Design	Invention	Control	NCT NO	Number of patients	Conditions	Lessons learned (intervention vs. control)
Markert et al. (110)	I Single Arm	G207 RT	NA	NCT00157703	9	rGBM	mOS 7.5 months
Todo et al. (114)	I/II Single Arm	G47Δ	NA	UMIN000002661	13	rGBM	mOS 7.3 months ^#^ ;1-year survival rate 38.5%
Todo et al. (115)	II Single Arm	G47Δ	NA	UMIN000015995	19	resGBM,rGBM	mOS 20.2 months 1-year survival rate 84.2%
Nassiri et al. (119)	I/II Single Arm	DNX-2401	NA	NCT02798406	49	rGBM	mOS 12.5months;12-month overall survival rate 52.7%;
Fares et al. (120)	I Single Arm	CRAd-S-pk7,SOC	NA	NCT03072134	12	11ndGBM,1AA	mOS 18.4 months; mPFS 9.05months
Chiocca et al. (127)	I Single Arm	Ad–RTS–hIL-12 DTX	ECP	NCT02026271	31	rGBM	mOS 12.7 months
Umemura et al. (124)	I Single Arm	Ad-hCMV-TK,Ad-hCMV-Flt3L,SOC	NA	NCT01811992	18	14GBM,13GS,1AE	mOS 21.3 months
Desjardins et al. (176)	I Single Arm	PVSRIPO	ECP	NCT01491893	61	recurrent grade IV glioma	mOS 12.5 vs. 11.3months

rGBM, recurrent glioblastoma; ndGBM, newly diagnosed glioblastoma; resGBM, residual glioblastoma; AA, anaplastic astrocytoma; GS, gliosarcoma; AE, anaplastic ependymoma; DTX, docetaxel; RT, radiotherapy; mOS, median overall survival; mPFS, median progression-free survival; SOC, Standard of Care, including surgical resection, concurrent chemoradiotherapy, adjuvant temozolomide for ndGBM; ECP, external control population; #, mOS is calculated from last administration, others without annotation are all calculated from randomization by default. All cited literature consists of peer-reviewed publications or registered clinical trial from the ClinicalTrials.gov database.

### 5.1 Oncolytic herpes simplex virus

Herpes simplex viruses (HSV) are neurotropic, double-stranded DNA viruses with large genomes (~150 kb) (104). Key oncolytic variants include HSV1716, HSV-G207, and G47Δ.

HSV1716, a first-generation oncolytic HSV, achieves tumor-selective replication through deletion of the RL1 gene (encoding ICP34.5), restricting viral propagation to actively dividing cells and thereby enhancing safety (105, 106). However, due to rapid tumor recurrence observed in Phase I trials following intracavitary infusion (107), HSV-G207 rapidly superseded HSV1716 in clinical development.

HSV-G207, a second-generation oncolytic herpes virus derived from HSV1716 through lacZ insertion-mediated inactivation of the ICP6 gene (UL39), exhibits restricted replication in normal cells by inhibiting late viral protein synthesis (108, 109). In a Phase I clinical trial for recurrent glioblastoma (rGBM),stereotactically guided intratumoral injection of HSV-G207 combined with a single 5 Gy radiotherapy fraction yielded a median overall survival of 7.5 months (110). The suboptimal therapeutic outcome likely reflects protocol limitations including use of a subtherapeutic radiation dose (5 Gy) and minimal therapeutic exposure (single viral injection plus single radiotherapy session), which collectively may have been insufficient to achieve adequate tumor cell destruction or elicit a robust antitumor immune response.

G47Δ, the third-generation oncolytic HSV-1, is engineered from the second-generation backbone by additionally inhibiting expression of the α47 gene in the G207 genome. This modification prevents downregulation of MHC class I molecules mediated through binding of the transporter associated with antigen presentation (TAP) in host cells (111, 112). Concurrently, it partially restores the function of the deleted γ34.5 gene, enhancing viral replication capability in tumor cells (113).

In the earliest phase I/II single-arm clinical trial conducted by Tomoki Todo's team, stereotactically guided intratumoral injection of G47Δ in 13 rGBM patients yielded a median survival of 7.3 months (114). However, subsequent analysis accounting for treatment parameters,including limited administration frequency (only 2 intratumoral infusions) and dose (3 patients received low-dose virus), revealed significantly improved outcomes in the latest phase II trial. The optimized protocol demonstrated a median survival of 20.2 months and 1-year survival rate of 84.2% in patients with rGBM or residual GBM (115).

### 5.2 Oncolytic adenovirus

Adenoviruses are non-enveloped, double-stranded DNA viruses that have been genetically modified for tumor-selective replication (116). Among the clinically evaluated oncolytic adenovirus platforms for glioblastoma treatment are DNX-2401, CRAd-S-pk7, and Onyx-015.

DNX-2401 in particular incorporates two key genetic modifications: a 24-base pair deletion in the E1A gene that restricts viral replication to cancer cells with aberrant retinoblastoma (Rb) pathway activity and insertion of an RGD-4C peptide motif in the fiber protein's HI loop to enhance tumor cell targeting through integrin binding (117, 118).

Phase I clinical trial results demonstrate that stereotactic intratumoral administration of DNX-2401 in combination with intravenous pembrolizumab yielded a median overall survival of 12.5 months in patients with rGBM (119), which represents a clinically meaningful improvement over historical monotherapy controls, suggesting synergistic therapeutic benefits from the combined viral immunotherapy and immune checkpoint inhibition approach.

CRAd-S-pk7 represents an innovative oncolytic adenovirus platform that utilizes neural stem cells (NSCs) as delivery vectors to penetrate the blood-brain barrier, while its tumor-specific survivin promoter restricts viral replication to survivin-overexpressing glioma cells. Phase I clinical trial data demonstrated promising efficacy in ndGBM, with the overall cohort achieving a median overall survival of 18.4 months in the treatment of the combination of intracavitary infusion of CRAd-S-pk7 and SOC, representing a 3.8-month improvement over SOC monotherapy, while the non-MGMT methylated subgroup showed particularly significant benefit with 18.0-month median survival (120).

### 5.3 PVSRIPO

PVSRIPO is an engineered oncolytic virus generated by replacing the poliovirus internal ribosomal entry site (IRES) with that of human rhinovirus type 2. This modification attenuates neurovirulence while preserving the natural CD155 tropism of poliovirus, enabling selective targeting of CD155-overexpressing tumor cells (121). Phase II clinical trial results demonstrated that convection-enhanced intratumoral delivery of PVSRIPO in recurrent glioblastoma (rGBM) patients yielded a median overall survival of 12.5 months - a significant improvement over historical controls (120). Based on these promising outcomes, an ongoing phase II trial is currently evaluating PVSRIPO in combination with pembrolizumab (NCT04479241).

### 5.4 Viral vectors

Viral vectors are engineered viral systems composed of modified structural elements from parent viruses (e.g. adenovirus, retrovirus) (122, 123) and therapeutic transgenes, such as immunomodulatory cytokines (IL-12, Flt3L), pro-apoptotic factors (Fas-L) and suicide genes (HSV-thymidine kinase) (124, 125), which are designed to safely deliver into target cells while maintaining efficient transduction capabilities and minimized pathogenicity.

IL-12 is a potent cytokine that enhances T-cell and natural killer (NK) cell activation/proliferation while exhibiting anti-angiogenic effects (126). The adenoviral vector Ad-RTS-hIL-12 carries an IL-12 expression cassette, enabling pharmacologically inducible IL-12 upregulation upon oral administration of veledimex (VDX). Phase I single-arm trial data demonstrated that intracavitary Ad-RTS-hIL-12 administration combined with oral veledimex (VDX) achieved a median overall survival of 12.7 months in patients with recurrent high-grade glioma (127).

The HSV-tk gene encodes thymidine kinase, which converts prodrugs (e.g., valacyclovir) into DNA synthesis-terminating metabolites (128). Flt3L stimulates dendritic cell development by binding Flt3 receptors on hematopoietic progenitors (129). In a Phase I trial for primary high-grade glioma, intracavitary delivery of adenoviral vectors (Ad-hCMV-TK + Ad-hCMV-Flt3L) combined with oral valacyclovir and SOC achieved a median overall survival of 21.3 months, warranting further validation (124).

In conclusion, as previously described, three delivery approaches are commonly utilized in oncolytic virus administration, including post-operative intracavitary infusion, stereotactically guided intratumoral injection for non-resectable lesions, and convection-enhanced delivery (CED) to achieve wider intraparenchymal distribution. The impact of pre-existing antiviral immunity remains a subject of active investigation,while low serum neutralizing antibody titers may theoretically permit enhanced viral spread due to reduced neutralization (130), they could alternatively indicate compromised host immune function that might limit therapeutic efficacy (130, 131). Besides, Radiographic assessment of treatment response presents unique challenges, as conventional criteria (e.g., RANO) may not fully capture virus-specific phenomena. For instance, G47Δ-treated tumors frequently demonstrate characteristic "exploded crater" morphology on MRI (114). These distinct imaging signatures underscore the need for revised response criteria specific to viral immunotherapies.

## 6 Challenges and countermeasures of GBM immunotherapy

**Figure 1:**
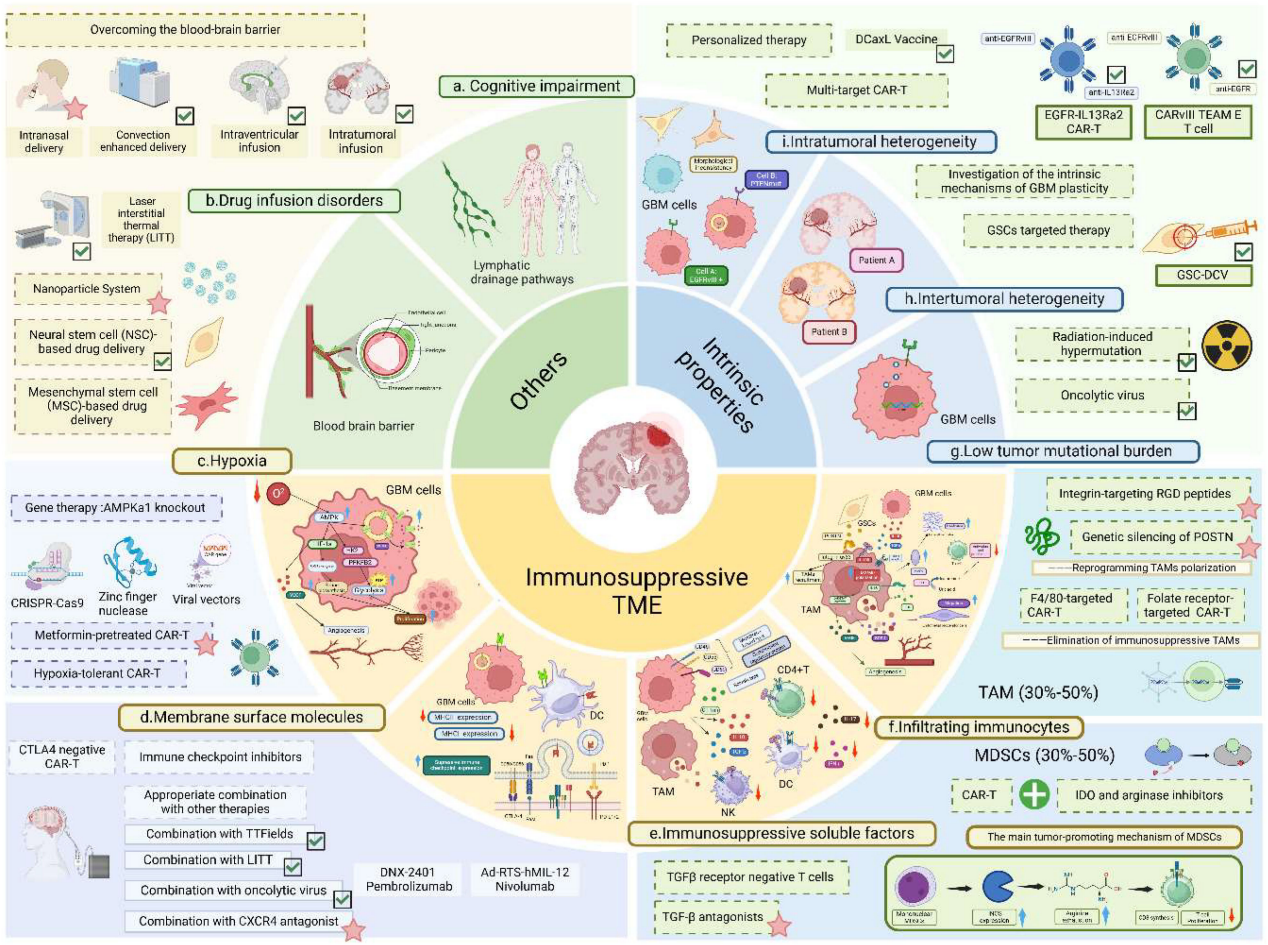
Challenges and countermeasures of glioblastoma immunotherapy. This figure provides a detailed illustration of the challenges in immunotherapy and corresponding countermeasures. GBM, glioblastoma multiforme; CAR-T, chimeric antigen receptor T-cell; DC, dendritic cell; NK, natural killer cell; TAM, tumor-associated macrophage; GSC, glioma stem cell; VEGF, vascular endothelial growth factor; SDF-1, stromal cell-derived factor-1;POSTN, periostin; IDO, indoleamine 2,3-dioxygenase; MMP9, matrix metalloproteinase-9; IL, interleukin; GSC-DCV glioma stem cell-derived dendritic cell vaccine; iNOS, inducible nitric oxide synthase. Detailed challenges: **(A)** Cognitive impairment of intracranial lymphatic drainage pathways; **(B)** Drug delivery obstacles due to the existence of blood-brain barrier; **(C)** Hypoxia.The hypoxic microenvironment upregulates intratumoral AMPK activity and mediates HIF-1α activation, thereby promoting glycolysis and glucose-derived *de novo* serine biosynthesis to facilitate tumor proliferation; **(D)** Membrane surface molecules. GBM cells evade immune surveillance by overexpressing inhibitory immune checkpoint ligands (e.g., PD-L1, Fas-L) and downregulating MHC expression; **(E)** Immunosuppressive soluble cytokines. Glioma cells express membrane-bound regulatory proteins (e.g., CD46, CD55, CD59) and secrete cytokines (e.g., IL-10, TGF-β) to suppress host immunity; **(F)** Infiltrating immune cells- GSCs recruit and polarize TAMs toward the M2 phenotype via secretion of POSTN and TGF-β. TAMs enhance tumor invasion by producing MMP9 to degrade the extracellular matrix, inhibit T-cell function via IDO1 expression, and promote angiogenesis by inducing VEGF secretion through the JAK-STAT pathway. Monocytic MDSCs suppress T-cell proliferation via iNOS expression; **(G)** Low tumor mutation burden; **(H)**. Intertumoral heterogeneity; **(I)** Intratumoral heterogeneity. Detailed countermeasures: Approaches including convection-enhanced delivery, intraventricular and intratumoral infusion, LITT, and neural stem cell-based delivery to overcome the blood-brain barrier; DCaxL and multi-targeted CAR-T to address tumor heterogeneity; radiotherapy, chemotherapy, and combined use of oncolytic viruses to tackle low tumor mutational burden; and the combination of immune checkpoint inhibitors with TTF and LITT to counteract T-cell suppression have been preliminarily validated in clinical trials (marked with green ticks). Meanwhile, methods including intranasal exosome delivery, AMPK kinase-preconditioned T cells, CXCR4 antagonists, and RGD peptides have shown promising results in preclinical GBM mouse studies (marked with pink stars). Additionally, strategies like mesenchymal stem cell-based drug delivery, low-oxygen-demand CAR-T, gene therapy targeting AMPKα1 knockout in tumor cells, CTLA4-negative CAR-T cells, F4/80-targeted CAR-T, and folate receptor-targeted CAR-T have demonstrated efficacy in preclinical studies of other cancers (e.g., ovarian cancer, lung cancer), offering valuable insights for the future development of GBM immunotherapy. The figure is created in BioRender.com.

Clinical trials of immunotherapies for glioblastomas are currently in full swing, but according to the above statement, most of the immunotherapies tend to be efficacious in early trials or single-arm studies, with few trials being able to demonstrate significant efficacy in phase III RCTs as they all face 4 main problems mentioned below-immunosuppressive tumor microenvironment, GBM intrinsic properties, drug infusion disorder and cognitive impairment of the lymphatic drainage pathways in the brain (17) (**Figure 1**).

### 6.1 immunosuppressive tumor microenvironment

The immunosuppressive TME comprises four key interrelated components: profound hypoxia, aberrantly expressed membrane surface molecules, immunosuppressive soluble factors and infiltrating immunocytes with regulatory functions.

#### 6.1.1 membrane surface molecules

Similar to other malignancies, glioblastoma (GBM) achieves immune escape through multiple pathways, comprising elevated expression of immune checkpoint ligands (e.g., PD-L1, Fas-L), reduced major histocompatibility complex (MHC) presentation and diminished co-stimulatory molecule expression (132–134). Beyond conventional immune checkpoint inhibitors, emerging strategies show promise: CTLA4-negative CAR-T cells have exhibited enhanced proliferative capacity and antitumor activity in leukemia patients, suggesting potential applicability to GBM (135). Checkpoint inhibitors combined with LITT/TTF has shown improved efficacy (42, 44) with more immunotherapy combinations mentioned below.

#### 6.1.2 Hypoxia

Hypoxic tumor microenvironment triggers AMPK activation in cancer cells, which stabilizes HIF-1α and subsequently induces the transcriptional upregulation of key metabolic regulators including glucose transporters (GLUT1, GLUT3 and GLUT4), glycolytic enzymes (HK2 and PFKFB2), and serine biosynthesis pathway (SSP) enzymes. These HIF-1α-mediated metabolic reprogramming events collectively enhance glycolytic flux and glucose-derived *de novo* serine biosynthesis, thereby promoting tumor cell proliferation (136–138). Paradoxically, this metabolic shift generates an immunosuppressive milieu through HIF-1α-mediated lactate accumulation, which impairs NK and T cell function (139). Preclinical studies demonstrate that metformin-pretreated CAR-T cells exhibit enhanced tumor infiltration and significantly prolong survival in glioma-bearing mouse models (140).Engineered hypoxia-tolerant CAR-T cells demonstrate potent antitumor activity with minimal toxicity in ovarian cancer models (141) and CRISPR/Cas9-mediated AMPKα1 knockout in lung cancer cells has been shown to generate stable cell lines with markedly reduced proliferation rates (142), both of which suggest potential translational relevance for GBM.

#### 6.1.3 Immunosuppressive soluble factors

Gliomas employ effectively shield themselves from complement-mediated destruction by expressing membrane-bound complement regulators like CD46, CD55, and CD59 (143). Simultaneously, their secretion of potent immunosuppressive cytokines such as IL-10 and TGF-β potently inhibits various immune populations including CD4+ T cells, NK cells, and dendritic cells leading to suppression of both antigen-specific and innate immune functions in patients (144, 145).

Recent preclinical advances highlight TGF-β inhibition as a particularly promising therapeutic avenue. When combined with radiotherapy, pharmacological TGF-β antagonists have been shown to reduce tumor invasiveness and reverse mesenchymal transition in GBM mice models, significantly extending survival (146). Equally encouraging, TGFBR2-knockout T cells demonstrate markedly enhanced tumor-killing capacity in both melanoma and lung adenocarcinoma models (147), providing valuable insights for its application in GBM treatment in the future.

#### 6.1.4 Infiltrating immunocytes with regulatory functions

By secreting periostin, GSCs recruit Tumor-Associated Macrophages (TAMs) into the tumor mass, where periostin-integrin αvβ3 binding initiates their pro-tumoral activation (148). Once recruited, these TAMs undergo functional reprogramming through sustained exposure to GSC-derived factors (TGF-β, IL-4, IL-10), adopting an immunosuppressive phenotype that actively supports tumor progression (149). The activated TAMs then facilitate tumor invasion through MMP9-mediated extracellular matrix degradation (150). Concurrently, they establish an immunosuppressive niche via IDO1-driven tryptophan metabolism, which inhibits effector T-cell function (151, 152). Furthermore, TAMs coordinate angiogenic processes through dual mechanisms: activating the IL-6/JAK-STAT/VEGF axis and secreting SDF-1 to recruit CXCR4+ endothelial progenitor cells (153, 154).

As is validated in preclinical trials, the CXCR4 antagonist plerixafor combined with anti-PD-1 (pembrolizumab) significantly improved survival of GBM mice (155); Treatment with integrin-targeting RGD peptides significantly suppressed POSTN-dependent TAM infiltration, resulting in reduction of tumor burden (148); Genetic silencing of POSTN in GSC-derived GBM models significantly prolonged overall survival (148); Both F4/80-targeted and folate receptor-directed CAR-T cells demonstrated significant tumor growth delay in ovarian cancer models (156, 157), which may be potentially applicable to GBM.

Clinically, combining CAR-T therapy with IDO/arginase inhibitors enhanced interferon production in solid tumors like colon cancer by blocking bone marrow-derived MDSC migration to tumor sites - a critical mechanism that prevents iNOS-mediated arginine depletion and subsequent impairment of CD3-TCR synthesis and T-cell proliferation (158, 159), which could be relevant for GBM therapy.

### 6.2 GBM intrinsic properties

The inherently low tumor mutational burden in glioblastoma creates a dual therapeutic challenge: it provides insufficient immune activation to overcome immune escape (160) while simultaneously restricting the pool of targetable neoantigens for immunotherapy development. Emerging strategies to enhance tumor immunogenicity include leveraging radiation-induced hypermutation (161, 162) or employing oncolytic viruses to stimulate TAAs release (163). Early clinical validation of these approaches is already emerging, with combination regimens such as DNX-2401 plus pembrolizumab (119) and Ad-RTS-hIL-12 with nivolumab (127) demonstrating preliminary efficacy in GBM patients.

Glioblastoma exhibits profound intratumoral and intertumoral heterogeneity, manifested through multiple dimensions. Molecular analyses reveal significant differences between the tumor core (TC) and peripheral brain tissue (PTB) regions (164). This variability is further compounded by treatment-induced antigen downregulation and phenotypic transformation in recurrent GBM (165). While molecular profiling initially classified GBM into three major subgroups (TCGA-PN, TCGA-CL, and TCGA-MES), most tumors demonstrate dynamic co-existence of these subtypes in varying proportions over time, underscoring the extensive spatial and temporal heterogeneity (3). Furthermore, GSCs serve as key drivers in establishing and maintaining this heterogeneous landscape (166).

Therapeutically, while multi-targeted approaches such as CART-EGFR-IL13Rα2 cells have achieved measurable tumor reduction in clinical trials (167), and DC vaccines combined with anti-PD-1 therapy (nivolumab) show survival benefits in rGBM (NCT02529072) (168), these strategies face inherent limitations due to tumor plasticity. Ultimately, targeting the fundamental mechanisms governing GSC plasticity may be required to achieve durable therapeutic responses.

### 6.3 Drug infusion disorder

While the blood-tumor barrier (BTB) represents a modified version of the blood-brain barrier (BBB) with altered integrity in tumor-affected regions (169, 170), neuroimaging studies reveal critical spatial heterogeneity in its disruption. MRI analyses demonstrate that in both glioblastoma (GBM) and medulloblastoma patients, BBB breakdown primarily localizes to the tumor core, while peritumoral areas often retain partial or even complete barrier functionality (171, 172). This persisting barrier function in infiltrative tumor regions necessitates targeted strategies to overcome residual drug delivery obstacles.

Emerging preclinical evidence highlights the promise of intranasal delivery platforms for treating central nervous system pathologies. In glioma models, curcumin-encapsulated exosomes administered intranasally significantly enhanced tumor cell apoptosis and improved survival in GL26-bearing mice (173), while engineered microglial exosomes expressing anti-LAG3 antibodies synergized with laser irradiation to boost glioblastoma responses to immune checkpoint inhibitors without developing resistance (174). In addition, mesenchymal stem cell (MSC)-based drug delivery systems have demonstrated efficacy in modulating neuroinflammatory processes in Alzheimer's disease models (175), suggesting their potential utility in transporting anti-tumor drugs.

## 7 Discussion

Glioblastoma (GBM) remains the most aggressive primary brain tumor despite multimodal therapeutic approaches including radiotherapy, TMZ chemotherapy, DNA damage response (DDR) inhibitors, and targeted molecular therapies. When it comes to immunotherapy, we must acknowledge the fact that while numerous previous immunotherapeutic agents have demonstrated remarkable survival benefits in early-phase clinical trials and single-arm studies, very few have shown efficacy in Phase III randomized controlled trials. Nevertheless, through optimization of drug design (such as continuously advanced oncolytic viruses and upgraded CAR-T cells), appropriate implementation of combination strategies and innovative treatment paradigms ( such as the appearance of neoadjuvant therapy) continue to make immunotherapy a promising approach for GBM patients, especially recurrent ones (**Tables 5**, **6**).

**Table 5:** Summary of promising drugs of immunotherapy for newly diagnosed patients.

Medication	Classification	Status	Stage	Design Type	NCT	n	mOS	95%Cl
Studies with Unlimited MGMT Methylation Status								
RT,TMZ(SOC)	Standard	ndGBM	III	RCT	NA	287	14.6	(13.2,16.8)
TTF,SOC	Standard	ndGBM	III	RCT	NCT00916409	466	20.9	(19.3,22.7)
SurVaxM,SOC	Vaccine	ndGBM	II	Single Arm	NCT02455557	63	25.9	(22.5,29)
PEM,TTF,SOC	ICI	ndGBM	II	Single Arm	NCT03405792	26	24.8	NA
Ad-hCMV-TK,Ad-hCMV-Flt3L,SOC	Virus	primary high-grade glioma	I	Single Arm	NCT01811992	18	21.3	(11.1,26.1)
DCVax-L,SOC	Vaccine	ndGBM	III	Single Arm	NCT00045968	232	19.3	(17.5,21.3)
IMA950,SOC	Vaccine	ndGBM	I/II	Single Arm	NCT01920191	16	19	(17.25,27.87)
CRAd-S-pk7, SOC	Virus	ndGBM	I	Single Arm	NCT03072134	12	18.4	(15.7, NA)
Studies in MGMT-Methylated Patients								
SOC	Standard	ndGBM	III	RCT	NCT01149109	63	31.4	(27.7,47.1)
LOM,SOC	Standard	ndGBM	III	RCT	NCT01149109	66	48.1	(32.6,NA)
DCVax-L, SOC	Vaccine	ndGBM	III	Single Arm	NCT00045968	90	30.2	(23.7,33.9)
SurVaxM, SOC	Vaccine	ndGBM	II	Single Arm	NCT02455557	33	41.4	(32.1,49.4)
Studies in MGMT-Methylated Patients								
SurVaxM, SOC	Vaccine	ndGBM	II	Single Arm	NCT02455557	28	16.5	(13.4,19.3)
CRAd-S-pk7, SOC	Virus	ndGBM	I	Single Arm	NCT03072134	9	18	(13.67,NA)

RCT, randomized controlled trial; TMZ, temozolomide; ndGBM, newly diagnosed glioblastoma; mOS, median overall survival; SOC, Standard of Care, including surgical resection, concurrent chemoradiotherapy, adjuvant temozolomide for ndGBM; RT, radiotherapy; TTF, tumor treating fields; IPI, ipilimumab; PEM, pembrolizumab; LOM, lomustine; ICI, immune checkpoint inhibitors. All cited literature consists of peer-reviewed publications or registered clinical trials from the ClinicalTrials.gov database.

**Table 6:** Summary of promising drugs of immunotherapy for recurrent patients.

Medication	Classification	Status	Stage	Design Type	NCT	n	mOS	95%Cl
Studies with Unlimited MGMT Methylation Status								
TMZ	Standard	rGBM	II	RCT	NCT00941460	53	10.6	(8.1,11.6)
BEV	Standard	rGBM	II	RCT	NCT00345163	85	9.2	(8.2, 10.7)
LOM	Standard	rGBM	III	RCT	NCT01290939	149	8.6	(7.6,10.4)
LOM+BEV	Standard	rGBM	III	RCT	NCT01290939	288	9.1	(8.1-10.1)
PEM(Neoadjavant)	ICI	rGBM	II	Single Arm	NCT02337686	15	20.3	(8.64,28.45)
G47Δ	Virus	resGBM, rGBM	II	Single Arm	UMIN000015995	19	20.2	(16.8,23.6)
NIVO	ICI	rGBM	III	RCT	NCT02017717	31	14.7	(8.9,17.2)
DCVax-L	Vaccine	rGBM	III	Single Arm	NCT00045968	64	13.2	(9.7,16.8)
Ad–RTS–hIL-12,DTX	Virus	rGBM	I	Single Arm	NCT02026271	31	12.7	NA
DNX-2401,PEM	Virus	rGBM	I	Single Arm	NCT02798406	49	12.5	(10.7,13.5)
PVSRIPO	Virus	recurrent grade IV glioma	I	Single Arm	NCT01491893	61	12.5	(9.9-15.2)
PEM,LITT	ICI	rGBM	II	RCT	NCT02337686	28	11.4	NA
HER2-CAR VSTs	CAR-T	rGBM	I	Single Arm	NCT01082926	17	11.1	(4.1,27.2)
IL-13Ra2 CAR-T	CAR-T	rGBM	I	Single Arm	NCT02208362	14	10.2	(7.7,NA)

RCT, randomized controlled trial; TMZ, temozolomide; BEV, bevacizumab; NIVO, nivolumab; DTX, docetaxel; LITT, laser interstitial thermal therapy; ndGBM, newly diagnosed glioblastoma; mOS, median overall survival; SOC, Standard of Care, including surgical resection, concurrent chemoradiotherapy, adjuvant temozolomide for ndGBM; PEM, pembrolizumab; LOM, lomustine; ICI, immune checkpoint inhibitors. All cited literature consists of peer-reviewed publications or registered clinical trials from the ClinicalTrials.gov database.

Notably, as diagnostic criteria evolution has introduced prognostic complexities: the 2021 WHO classification now defines GBM specifically as IDH-wildtype (IDHwt) tumors (5), earlier clinical trials, however, included IDH-mutant cases, potentially yielding more optimistic survival outcomes than those expected in trials conducted under current diagnostic standards. This historical discrepancy underscores the importance of molecular stratification in interpreting therapeutic results across different eras of GBM research. Furthermore, since tumor-treating fields were only formally incorporated into the SOC for glioblastoma in the 2021 WHO Classification of Central Nervous System Tumors (5), the control arms across cited in clinical trials consistently employed conventional SOC regimens consisting of concurrent chemoradiotherapy followed by adjuvant TMZ therapy.

In summary, the effectiveness of immunotherapy in glioblastoma is limited by several key factors, including the tumor's low mutational burden, its highly heterogeneous nature, the strongly immunosuppressive microenvironment, drug delivery obstacles, and the brain's unique lymphatic anatomy.

To counteract the hypoxic tumor microenvironment, potential approaches include using gene therapy to knock down AMPKα1 in tumor cells or pretreating T cells with AMPK kinase activators, developing CAR-T cells with low oxygen requirements. For GBM’s escape immunity by downregulating TGFβ receptors while overexpressing TGFβ, solutions involve creating TGFβ receptor-negative T cells or administering TGFβ antagonists. The recruitment of tumor-associated macrophages by POSTN might be blocked using integrin-inhibiting RGD peptides, while CAR-T cells targeting F4/80 and folate receptors could directly eliminate TAMs.

To improve drug delivery, potential strategies include intranasal administration, convection-enhanced delivery, direct tumor or ventricular infusion, and laser interstitial thermal therapy (LITT). Alternative approaches using neural stem cells or nanoparticles (including exosomes) may help drugs cross the blood-brain barrier.

Addressing glioblastoma's fundamental characteristics requires more than just increasing mutational load through radiotherapy or oncolytic viruses. The core challenge lies in overcoming the tumor's subclonal diversity and poor immunogenicity, which are critical for generating effective anti-tumor immune responses.

Beyond the inherent biological and technical hurdles in treating glioblastoma (GBM), the clinical implementation of immunotherapies faces critical logistical and financial barriers that demand urgent attention. Logistically, the cold-chain dependence and time-sensitive nature of many immunotherapeutic agents (e.g., CAR-T cells) pose significant supply-chain challenges. Potential solutions include accelerating the development of universal CAR-T products to reduce manufacturing delays and advancing lyophilized formulations (e.g., BioNTech's room-temperature-stable RNA vaccine technology) to minimize cold storage requirements.

Financially, the prohibitive costs of GBM immunotherapy remain a major obstacle, driven by both the expensive personalized production of immunotherapies (e.g., tumor-specific peptide vaccines) and limited insurance coverage for most emerging treatments. To address affordability, broader adoption of risk-sharing agreements (e.g., the UK NHS's "pay-for-performance" model) and global funding initiatives (e.g., WHO's immunotherapy accessibility programs) could help alleviate the economic burden on patients and healthcare systems.
